# Investigating the Role of Ferritin in Determining Sexual Underdevelopment in Beta-Thalassemia Major Patients: A Cross-Sectional Analysis From Pakistan

**DOI:** 10.7759/cureus.15572

**Published:** 2021-06-10

**Authors:** Zia Shahid, Sarmad Hassan, Saqlain Ghazanfar, Mehwish Kaneez, Muhammad Sheharyar Khan, Hafiz Tanveer Tariq, Arslan Jawad, Atifa Shuaib, Assadullah Akram Bhatti, Mustafa Tauseef Razzaq

**Affiliations:** 1 Internal Medicine, Rawalpindi Medical University, Rawalpindi, PAK; 2 Pediatrics, Rawalpindi Medical University, Rawalpindi, PAK; 3 Internal Medicine, Hamad Medical Corporation, Doha, QAT; 4 Pathology, Rawalpindi Medical University, Rawalpindi, PAK; 5 Internal Medicine, Rashid Latif Medical College, Lahore, PAK

**Keywords:** sexual underdevelopment, beta-thalassemia, ferritin, iron overload, tanner staging system

## Abstract

Background

Beta-thalassemia major, a genetic disorder, delineates a vast spectrum of hematological and endocrinological complications. Elevated serum ferritin levels in beta-thalassemia patients represent various transfusion-related complications including infection, hemochromatosis, and severe iron overload that might lead to endocrinopathies such as hypogonadism leading to sexual underdevelopment. Our study, thus, aims to explore the role of ferritin in determining sexual underdevelopment in such patients.

Methods

This multicentric cross-sectional study included a total of 120 beta-thalassemia patients. The sexual development of the patients was assessed using the Tanner staging system. Serum ferritin levels and other demographical parameters of the patients were collected. Independent-samples t-test, chi-square test, and receiver operating characteristic (ROC) curve were used to analyze the data.

Results

Out of 120 patients, 70 patients were males with a mean age of 18.95 ± 4.21 years. According to the Tanner staging system, 48 patients were sexually underdeveloped while 72 patients achieved sexual maturity. ROC curve analysis showed that ferritin levels at a cutoff value of 4900 mg/dL were 73.7% sensitive and 71.1% specific to predict sexual underdevelopment in beta-thalassemia patients.

Conclusions

Elevated serum ferritin levels were moderately sensitive and specific in predicting sexual underdevelopment in beta-thalassemia patients. This can serve as a low-cost parameter in determining sexual underdevelopment in such patients. More prospective cohort studies are needed to establish the association between elevated serum ferritin levels and sexual underdevelopment.

## Introduction

Beta-thalassemia major is a genetic disorder in the beta-globin gene that encompasses a vast array of hematological manifestations that ultimately translate into microcytic anemia [1]. These patients require frequent blood transfusions or bone marrow transplantation for their survival [1,2]. Around the world, nearly four of every 100,000 live births are affected with beta-thalassemia and a vast number are asymptomatic carriers of the affected gene [2]. Statistics from Pakistan indicate that approximately 70,000 patients are suffering from beta-thalassemia major while 6000 patients are registered for the treatment of beta-thalassemia, and the number is rising exponentially [3].

Literature shows that during the 1950s, less than 9% of the beta-thalassemia patients reached the age of six years, while in the 1970s more than half of the Italian thalassemic patients had died before reaching 12 years of age [4]. However, a major improvement was observed in the survival rate of these patients due to the introduction of combined safe transfusions, regular chelation, and bone marrow transplantation [1-4]. Regular and multiple blood transfusions are essential for the survival of beta-thalassemia; however, it ultimately translates into severe iron overload [5]. After 15-20 blood transfusions, the risk of secondary hemochromatosis is significantly elevated among such patients [6]. This iron overload creates havoc in the body that exceeds the binding capacity of ferritin and transferrin leading to free iron circulation in the plasma. This free iron leads to the formation of different radicals resulting in complications including various endocrinopathies [5,6].

Studies have shown an alarmingly high mortality rate in beta-thalassemic patients with increased ferritin levels [7,8]. Similarly, beta-thalassemia has also been associated with various endocrinopathies (diabetes, hypopituitarism, hyperparathyroidism, hypogonadism, and hypothyroidism) including hypogonadism, which might lead to delayed sexual development [8,9]. Hypogonadism is a frequent complication that occurs secondary to iron deposition in the hypothalamus, pituitary gland, and gonads [10,11]. Although in-time use of chelating drugs like deferoxamine can bring improvement in survival, it is a great cause of concern for the patients as well as the pediatricians [12].

One of the standard tools used for the assessment of sexual development is the Tanner staging system that is a simple and inexpensive method that is commonly utilized for documenting the development of secondary sexual characteristics during puberty [13]. Our study, thus, aims to explore the role of ferritin in determining the sexual underdevelopment in beta-thalassemia patients while using the Tanner staging system as standard. It will aid in appraising the significance of chelation therapy and declining ferritin levels to certain extent that may improve the quality of life of beta-thalassemia major patients.

## Materials and methods

This multicentric cross-sectional study included a total of 135 beta-thalassemia major patients registered in Holy Family Hospital, Jamila Sultana Foundation, and Pakistan Institute of Medical Sciences. These three centers were from Rawalpindi and Islamabad, Pakistan. The data collection was performed from March 2018 to May 2019 using a convenience sampling technique. All male and female patients with age between 13 and 30 were included in the study. Patients with comorbidities (diabetes, hypothyroidism, or metabolic disorders) or having an underlying infectious or inflammatory disease were excluded from the study. Furthermore, patients who did not agree to participate in the study were also excluded. After the application of the exclusion criteria, 120 patients qualified for the final analysis.

Written informed consent was taken from the patients and their parents and the sexual development of the patients was assessed using the Tanner staging system. The sexual maturation of the patients was classified according to the pubertal staging on the Tanner scale [13]. Boys were assessed for genital development and pubic hair growth, and girls were evaluated for breast development and pubic hair growth. On Tanner staging, patients at the pre-pubescent stage (stage 1 or stage 2) were considered sexually underdeveloped. In contrast, patients at the pubescent (stage 3) or post-pubescent (stages 4 and 5) stages were considered sexually developed. After consent from the parents and patients, a senior doctor examined the children for their respective stages of secondary sexual development. The ethical approval was granted from the institutional research forum of Rawalpindi Medical University, Rawalpindi, Pakistan with approval number 07-PM-2019.

The results of Tanner staging were compared with various laboratory parameters (serum ferritin levels and hemoglobin levels) and demographical variables (age, height, and, weight). Independent-samples t-test was used for this comparison. Thereafter, the receiver operating characteristic (ROC) curve was plotted to assess the predictive power of ferritin for sexual underdevelopment. A p-value of <0.05 was deemed statistically significant and data analysis was carried out on Statistical Package for Social Sciences (SPSS, IBM, Armonk, NY, USA) version 23.0.

## Results

A total of 120 participants were examined, of which 70 (58.3%) were males and 50 (41.7%) were females. The mean age of the study participants was 18.95 ± 4.21 years and ranged between 13 and 30 years. According to the Tanner staging system, 48 (40%) of the patients were in the pre-pubescent stage, while 72 (60%) of the patients were in the pubescent and post-pubescent stage. This distribution of the patients is shown in Table 1.

**Table 1:** A breakdown of Tanner staging among the study participants

Tanner staging	Frequency	Percentage	Sexually underdeveloped
Pre-pubescent stage	48	40.0	Yes
Pubescent stage	40	33.3	No
Post-pubescent stage	32	26.7	No

Out of 48 sexually underdeveloped patients, 18 were males and 30 were females. The characteristics of the study participants were stratified based on sexual underdevelopment, which yielded a statistically significant difference in the means of age, height, weight, recent ferritin, and hemoglobin levels as elucidated in Table 2.

**Table 2:** A comparison between sexually underdeveloped and sexually developed patients *Independent-samples t-test

Parameters	Sexual underdevelopment		p-Value*
	Yes	No	
	Mean ± standard deviation	Mean ± standard deviation	
Age of the patient (years)	16.4 ± 2.2	21.1 ± 4.4	0.001
Height of the patient (cm)	141.5 ± 6.1	155.7 ± 16.6	0.002
Weight of the patient (kg)	37.3 ± 6.1	43.4 ± 8.4	0.001
Age at start of transfusion treatment (months)	11.9 ± 1.1	13.2 ± 1.7	0.603
Ferritin levels (mg/dL)	5907 ± 2697	4165 ± 2982	0.003
Hemoglobin level (g/dL)	7.9 ± 1.8	8.8 ± 1.3	0.006

The ROC curve analysis of ferritin to predict sexual underdevelopment is shown in Table 3.

**Table 3:** Sensitivity and specificity of ferritin levels to predict sexual underdevelopment

Parameter	Area	95% Confidence interval	p-Value	Selected cutoff value	Sensitivity at cutoff	Specificity at cutoff
Ferritin levels	0.709	0.605-0.813	0.001	4900 mg/dL	73.7%	71.1%

## Discussion

Various endocrine abnormalities (hypothyroidism, hypopituitarism, and diabetes) have been associated with serum iron overload due to multiple blood transfusions in beta-thalassemia patients [1,7]. Of these endocrine abnormalities, hypogonadotropic hypogonadism resulting from iron overload is regarded as the most evident endocrinopathy that impairs the quality of life of beta-thalassemia patients [11].

According to a study conducted in Iran, beta-thalassemia patients with elevated serum ferritin levels were two to four times more prone to develop cardiac or hepatic iron overload, which can result in significant mortality [7]. The most frequent cause of mortality in such patients includes transfusion-transmitted infections, severe iron overload in vital organs, and alloimmunization [8,10]. Our study shows that sexually underdeveloped patients had higher ferritin levels than patients with complete sexual development. This shows that severe iron overload might be the reason for sexual underdevelopment. These findings can be validated with many studies that displayed a direct association between serum ferritin levels and the presence of different endocrinopathies, namely diabetes mellitus, hypothyroidism, hypoparathyroidism, and hypogonadism [8,9]. Similarly, a study conducted in Turkey necessitated the need for frequent follow-up for various endocrinopathies in beta-thalassemia patients with increased serum ferritin levels [9].

Even though beta-thalassemia illustrates a poor disease progression, the development of safer transfusion practices along with adjuvant chelation therapy have profoundly improved the life expectancy of beta-thalassemic patients [1,3]. However, the growth spurt and sexual development are often delayed, if timely intervention with intensive iron chelation therapy is not performed [6]. In our study, we compared sexual underdevelopment based on certain attributes and parameters. The height of sexually underdeveloped patients was significantly lower than the height of patients with normal sexual development. A study conducted from Greece also reports similar findings [14]. This growth retardation is due to the decreased levels of growth hormone associated with increased ferritin levels [15]. Another parameter assessed for sexually underdeveloped beta-thalassemia patients was weight. It was found that the weight of the sexually underdeveloped patients was also lower than their counterparts. The results of another study validate our finding [16].

Primarily, we performed an ROC curve analysis to evaluate the sensitivity and specificity of ferritin for the assessment of sexual underdevelopment. We found that a certain cutoff value of 4900 mg/dL had a sensitivity and specificity of 73.7% and 71.1%, respectively. Literature reports different methods of assessing hypogonadism in thalassemia patients. These include MRI, levels of follicle-stimulating hormone, luteinizing hormone, estradiol, and testosterone [15,17]. However, these diagnostic methods are very time-consuming and expensive for a resource-deprived healthcare system of a developing country like Pakistan where the prevalence of beta-thalassemia is increasing [3,7]. There is an urgent need to formulate an abrupt and easy laboratory parameter to monitor sexual underdevelopment in beta-thalassemia patients. Thus, the present study complies with the conditions laid down and provides a parameter with reasonable sensitivity and specificity that is routinely assessed in patients of beta-thalassemia. 

Hypogonadotropic hypogonadism can be successfully treated with iron chelation and hormone replacement therapy (HRT) with regular monitoring [17]. The treatment aims to maintain fertility, secondary sexual characteristics, and not delay the onset of puberty. Moreover, it also aims to prevent disease complications and improve the quality of life. Deferiprone and deferoxamine when used together have been reported to improve sexual development in thalassemia patients [15]. Furthermore, serum ferritin levels can also be used to regularly monitor the effectiveness of chelation therapy. However, HRT has been linked with many complications; hence, it should be used cautiously in thalassemia patients [18].

A cross-sectional study design and use of convenience sampling technique account for the limitations of our study. Nonetheless, our study highlights the need for conduction of further researches of prospective nature so that the association of serum ferritin levels and sexual underdevelopment can be further explored. Our study opens up new horizons for future research. As we evaluated the role of ferritin for sexual underdevelopment, the role of other hematological and inflammatory markers such as C-reactive protein (CRP) should also be evaluated. Similarly, the ferritin-to-CRP ratio may also be used to determine sexual underdevelopment. This will aid in the development of relatively inexpensive laboratory parameters for evaluation of sexual underdevelopment in beta-thalassemia major patients.

## Conclusions

Sexual underdevelopment is a cause of serious concern in beta-thalassemia patients as it represents the delayed onset of puberty. Serum ferritin levels of more than 4900mg/dL have moderate sensitivity and specificity in predicting sexual underdevelopment in such patients. As serum ferritin levels are routinely evaluated in patients with beta-thalassemia, it can serve as an important laboratory parameter for the assessment of sexual underdevelopment in beta-thalassemia patients. We recommend further case-control and prospective cohort studies on the topic to investigate the role of serum ferritin and iron overload in predicting sexual underdevelopment in beta-thalassemia patients.
